# A General System of Gardening and Botany; Containing a Complete Enumeration and Description of All Plants Hitherto Known; with Their Generic and Specific Characters, Places of Growth, Time of Flowering, Mode of Culture, and Their Uses in Medicine and Domestic Economy. Preceded by Introductions to the Linnæan and Natural Systems, and a Glossary of the Terms Used. Founded upon "Miller's Gardener's Dictionary," and Arranged According to the Natural System

**Published:** 1833-01

**Authors:** 


### BIBLIOGRAPHICAL NOTICE.

*A General System of Gardening and Botany; containing a complete Enumeration and Description of all Plants hitherto known; with their Generic and Specific Characters, Places of Growth, Time of Flowering, Mode of Culture, and their Uses in Medicine and Domestic Economy. Preceded by Introductions to the Linnæan and Natural Systems, and a Glossary of the Terms used. Founded upon "Miller's Gardener's Dictionary," and arranged according to the Natural System.* By George Don, f.l.s. In four volumes, 4to. Vols. I. and II. — Pp. 818 and 875; with figures on wood. Rivingtons, &c.

Did not the title-page profess, and the advertisements declare, this work to be founded upon "Miller's Gardener's Dictionary," of which it was at first intended to have been a new edition, we confess that we should not very readily have guessed at the foundation; for it is no more founded upon the Dictionary of Miller than it is upon the Prodromus of De Candolle, and scarcely so much so; and it might, without any suspicion of plagiary, have been ushered into the world as an original work by the present editor (George Don), whose name would have been a sufficient guarantee of its worth, even without the sanction of Miller's popularity. But, as the proprietors and publishers, who are shrewd philosophers, as far as mercantile literature is concerned, have judged the present title to be the most advantageous, it is our duty rather to pay attention to the subsequent pages than to criticise the first.

As to reading these two quarto volumes, we make no pretensions to have done so; for who ever read a Dictionary, except to revise the proofs? but we have opened them in numerous places, and have made many references to their columns, and we are enabled to speak very favorably of their contents.

A complete English Species Plantarum, brought up to the present day, was much wanted, and this Mr. Don promises to supply as far as the Ferns. Had the object of this work been to form an analytic scale, our approbation of the plan must have been, however unwillingly, withheld; but, as a history of plants, the scheme of natural affinities is decidedly the best to be pursued. The facility, likewise, of a dictionary reference is gained by the alphabetical index prefixed to each volume, and, we should hope, would be still further insured by a general index to the whole.

In the second volume, the editor has inserted *entire* the catalogues of the varieties of pear and apple, which are but just published by the Horticultural Society. This appropriation of their labours roused the ire of the Fellows, and an injunction against the sale of the volume was applied for, and obtained. We think it would have been but courteous in Mr. Don to have asked permission to copy the catalogue; but we think it far below the objects of literary associations to be so very jealous of their trading concerns. The Transactions of learned Societies are always regarded as public property, and, the more use can be made of them, the more fully are the purposes of such institutions attained: we are, therefore, glad that the injunction has been withdrawn. Still we think it would have more honourable if the Society had not noticed the circumstance, than, without any benefit even in a pecuniary point of view to themselves, to have thus put the editor to the expense of forty pounds, and, after shewing the *malus animus*, to have feared to perpetrate the *mala acta.*

In the introduction, it is proposed to conclude this work with the Ferns. We trust that decision is not irrevocable; or, if it does resemble the laws of the Medes and Persians, which alter not, that Mr. Don will be persuaded to complete the species of the vegetable kingdom, by publishing an appendix, in which the Mosses, Fungi, and Algæ may appear.

On looking through these two volumes, forming half the space allotted to the work, we find that so much less than half of the task proposed has been completed, that we think it impossible it can be finished in four volumes: we trust that the learned editor will not suffer a desire of not exceeding that space to lead him to condense unduly the remaining portion of his labours.

The editor states that THE natural system is followed in this work: we should have felt obliged if he had informed us what natural system; for, as so many THE natural systems are in vogue, we think the indefinite more fitting than the definite article. We, however, suppose that it is De Candolle's version here adopted, with variations; for it certainly is not Jussieu's. In the Jussieuan arrangement, the simplest plants were those the first described, and the series ran from these through more and more elaborate developments; but why De Candolle, Don, and others, commence with Ranunculaceæ, and why to Clematis all other plants should yield the post of honour, we never could divine.

